# Significantly enhanced dye removal performance of hollow tin oxide nanoparticles via carbon coating in dark environment and study of its mechanism

**DOI:** 10.1186/1556-276X-9-442

**Published:** 2014-08-28

**Authors:** Shuanglei Yang, Zhaohui Wu, LanPing Huang, Banghong Zhou, Mei Lei, Lingling Sun, Qingyong Tian, Jun Pan, Wei Wu, Hongbo Zhang

**Affiliations:** 1 State Key Laboratory for Powder Metallurgy, Central South University, Changsha 410083, People's Republic of China; 2 Laboratory of Printable Functional Nanomaterials and Printed Electronics, School of Printing and Packaging, Wuhan University, Wuhan 430072, People's Republic of China; 3 Key Laboratory of Artificial Micro and Nano-structures of Ministry of Education, School of Physics and Technology, Wuhan University, Wuhan 430072, People's Republic of China; 4 Department of Chemical Engineering, Kyung Hee University, Seocheon-Dong, Giheung-Gu, 446-701 Yongin, Korea

**Keywords:** Tin oxide, Carbon coating, Core-shell, Dye removal

## Abstract

Understanding the correlation between physicochemical properties and morphology of nanostructures is a prerequisite for widespread applications of nanomaterials in environmental application areas. Herein, we illustrated that the uniform-sized SnO_2_@C hollow nanoparticles were large-scale synthesized by a facile hydrothermal method. The size of the core-shell hollow nanoparticles was about 56 nm, and the shell was composed of a solid carbon layer with a thickness of 2 ~ 3 nm. The resulting products were characterized in terms of morphology, composition, and surface property by various analytical techniques. Moreover, the SnO_2_@C hollow nanoparticles are shown to be effective adsorbents for removing four different dyes from aqueous solutions, which is superior to the pure hollow SnO_2_ nanoparticles and commercial SnO_2_. The enhanced mechanism has also been discussed, which can be attributed to the high specific surface areas after carbon coating.

## Background

With the development of society and scientific technology, more attentions have been paid to environmental issues which were caused by the discharge of wastewater. Oil spillage, organic solvents, and synthetic dyes discharged by the textile, paper, and tannery industries are primary pollutants of water sources [1]. It is estimated that more than 100,000 commercially available dyes with over 7 × 10^5^ tonnes of dyestuff are produced annually [2]. Generally, synthetic dyes have complex aromatic structures that make them stable and difficult to biodegrade. As synthetic dyes in wastewater cannot be efficiently decolorized by traditional methods (such as catalytic degradation, cation exchange membranes, and biological processes), the adsorption of synthetic dyes on inexpensive and efficient solid supports was considered as a simple and economical method for their removal from water and wastewater.

The most commonly used absorbent for dye removal is activated carbon, because of its capability for efficiently adsorbing a broad range of different types of dyes [3]. Up to now, there have been many successful methodologies for the fabrication of activated carbon materials, such as pinewood-based activated carbon [4], coir pith activated carbon [5], rice husk-based activated carbon [6], and bamboo-based activated carbon [7]. Although, natural renewable resources have been widely used as raw materials for manufacturing activated carbon, the high production and treatment costs of activated carbon may still hinder its further application.

As a competitive alternative, various nanomaterials have been developed and used to remove the dyes. For example, Zhu and co-workers have prepared hierarchical NiO spheres with a high specific area of 222 m^2^/g as an adsorbent for removal of Congo red [8]. Mou and co-workers have fabricated γ-Fe_2_O_3_ and Fe_3_O_4_ chestnut-like hierarchical nanostructures, which can be separated simply and rapidly from treated water by magnetic separation after As(V) adsorption treatment. And the As(V) removal capacity of as-obtained γ-Fe_2_O_3_ is maintained at 74% and reaches 101.4 mg/g [9]. And then, they have prepared magnetic Fe_2_O_3_ chestnut-like amorphous-core/γ-phase-shell hierarchical nanostructures with a high specific area of 143.12 m^2^/g and with a maximum adsorption capacity of 137.5 mg/g for As(V) adsorption treatment [10]. Liu and co-workers have prepared various bismuth oxyiodide hierarchical architectures, and their nanomaterials shown enhanced the photocatalytic performance and adsorption capabilities [11]. Recently, the carbon functionalized nanomaterials have recently attracted considerable attention because of their enhanced dye removal performance. For instance, Fan and co-workers have synthesized hybridization of graphene sheets and carbon-coated Fe_3_O_4_ nanoparticles as an adsorbent of organic dyes [12]. Li and co-workers have reported Mg(OH)_2_@reduced graphene oxide composite, which exhibited excellent adsorption behavior for methylene blue (MB) [13].

Indeed, the adsorption technique is especially attractive because of its simple design, high efficiency, and easy operation, but it requires materials with large specific surface area, well-defined pore size, and shape. Hollow structured materials fit these criteria well, and they have attracted tremendous interest as a special class of materials compared to other solid counterparts, owing to their higher specific surface area, lower density, and better permeation, which have been extensively considered as potential materials applied in adsorption, catalysis, chemical reactors, and various new application fields [14-16]. Therefore, design and fabrication of materials like carbon-coated hollow structure would increase the dye removal abilities.

Herein, a well-defined carbon-coated hollow SnO_2_ nanoparticles have been designed and fabricated by a facile two-step hydrothermal method without using any surfactants. Carbon coating prepared by hydrothermal treatment of low-cost glucose has aroused much interest. The preparation process belongs to green chemistry as the reaction process is safe and does not incur any contamination of the environment. More importantly, the carbon layer increases the specific area of bare hollow SnO_2_ nanoparticles, which exhibits an enhanced dye removal performance.

## Methods

### Materials

Potassium stannate trihydrate (K_2_SnO_3_ · 3H_2_O), commercial SnO_2_, rhodamine B (RhB), MB, rhodamine 6G (Rh6G), and methyl orange (MO) were purchased from Shanghai Jingchun Chemical Reagent Co., Ltd. (Shanghai, China). Urea (CO(NH_2_)_2_), ethylene glycol (EG), ethanol (C_2_H_5_OH), and glucose (C_6_H_12_O_6_) were purchased from Sinopharm Chemical Reagent Co., Ltd. (Shanghai, China). All the materials were used without further purification in the whole experimental process. Deionized water was used throughout the experiments.

### Synthesis of hollow SnO_2_ nanoparticles

In a typical process, 0.6 g potassium stannate trihydrate was dissolved in 50 mL ethylene glycol through the ultrasonic method. Urea (0.4 g) was dissolved in 30 mL deionized water and then the solution was mixed together and transferred into a Teflon-lined stainless steel autoclave with a capacity of 100 mL for hydrothermal treatment at 170°C for 32 h. The autoclave solution was removed from the oven was allowed to cool down to room temperature. The product was harvested by centrifugation and washed with deionized water and ethanol and then dried at 80°C under vacuum.

### Synthesis of hollow SnO_2_@C nanoparticles

SnO_2_@C hollow nanoparticles were prepared by a glucose hydrothermal process and subsequent carbonization approach. In a typical process, 0.4 g of as-prepared hollow SnO_2_ nanoparticles and 4 g glucose were re-dispersed in ethanol/H_2_O solution. After stirring, the solution was transferred into a 100-ml Teflon-lined stainless steel autoclave sealed and maintained at 170°C for 8 h. After the reaction was finished, the resulting black solid products were centrifuged and washed with deionized water and ethanol and dried at 80°C in air. Lastly, the black products were kept in a tube furnace at 600°C for 4 h under argon at a ramping rate of 5°C/min.

### Characterization

Transmission electron microscopy (TEM) and high-resolution transmission electron microscopy (HRTEM) were performed with a JEOL JEM-2100 F transmission electron microscope (Tokyo, Japan) at an accelerating voltage of 200 kV, and all the samples were dissolved in ethanol by ultrasonic treatment and dropped on copper grids. Powder X-ray diffraction (XRD) patterns of the samples were recorded on a D/ruanx2550PC (Tokyo, Japan) using CuKα radiation (*λ* = 0.1542 nm) operated at 40 kV and 40 mA. The absorption spectra of the samples were carried out on a Shimadzu UV-2550 spectrophotometer (Kyoto, Japan). Raman measurement was performed using a HORIBA Jobin Yvon LabRAM HR spectroscope (Edison, NJ, USA) using an excitation laser wavelength of 488 nm.

### Adsorption experiments

Of the samples, 5 mg was re-dispersed in 10 mL of the organic dyes (concentration 10 mg/L) and the mixed solution was stored in the dark for 45 min with gentle stirring. The reaction solution was sampled every 15-min intervals at room temperature; 2 mL solution was sampled and centrifuged to remove the adsorbents, and the corresponding UV-visible spectra were recorded to monitor the progress of the degradation of organic dyes by a Shimadzu 2550 UV-visible spectrophotometer.

## Results and discussion

**Figure 1:**
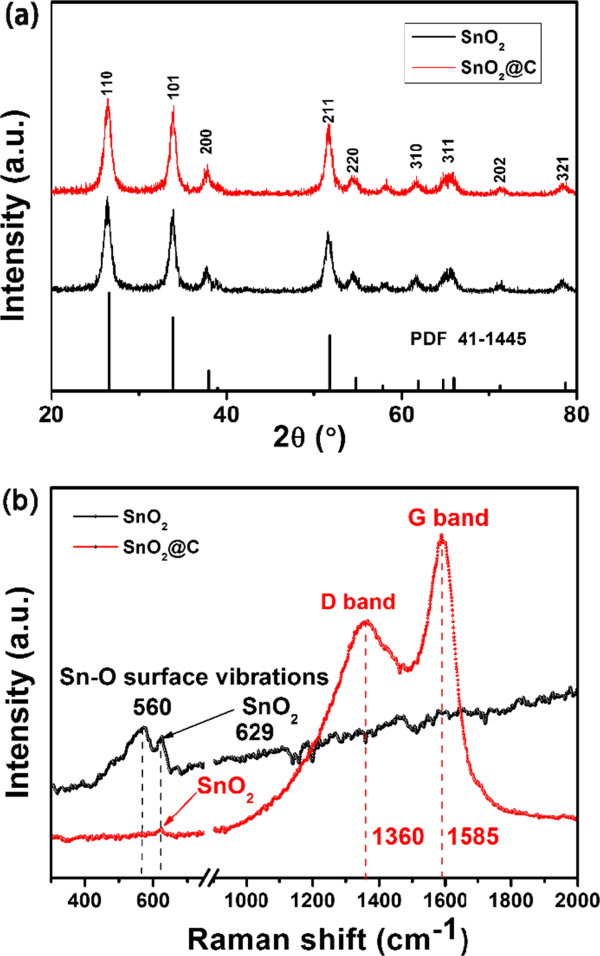
**XRD patterns (a) and Raman spectra (b) of the as-obtained hollow SnO**_**2 **_**and hollow SnO**_**2**_**@C nanoparticles.**

Figure 1a shows the representative XRD patterns of the as-obtained hollow SnO_2_ and hollow SnO_2_@C nanoparticles. All of the diffraction peaks can be well indexed to the tetragonal rutile phase of SnO_2_ (JCPDS card No. 41-1445). The absence of characteristic peaks corresponding to impurities indicates high purity of the products [17]. The result reveals that the carbon coating process and annealing treatment will not change the structure of the SnO_2_. To prove the generation of the carbon layer on the as-prepared hollow SnO_2_ seeds, the two samples were characterized by Raman spectroscopy. As shown in Figure 1b, the two peaks of 1,585 and 1,360 cm^−1^ can be observed in the hollow SnO_2_@C sample, which can be attributed to the E_2g_ vibration mode of the ordered carbon layer (G band) and the A_1g_ vibration mode of the disordered carbon layer (D band), respectively. The peak intensity ratio (*I*_D_/*I*_G_) (ca. 0.76) calculated is a useful index for comparing the degree of crystallinity of various carbon materials; a smaller value ratio reflects a higher degree of ordering in the carbon material. The peaks at 560 and 629 cm^−1^ can be observed, respectively. The peak at 560 cm^−1^ can be assigned to the Sn-O surface vibrations; the peak at 629 cm^−1^ can be indexed to the A_1g_ mode of SnO_2_. The above results reveal that the carbon has been successfully coated on the surface of the SnO_2_ nanoparticles, and the structure of SnO_2_ was not change.

**Figure 2:**
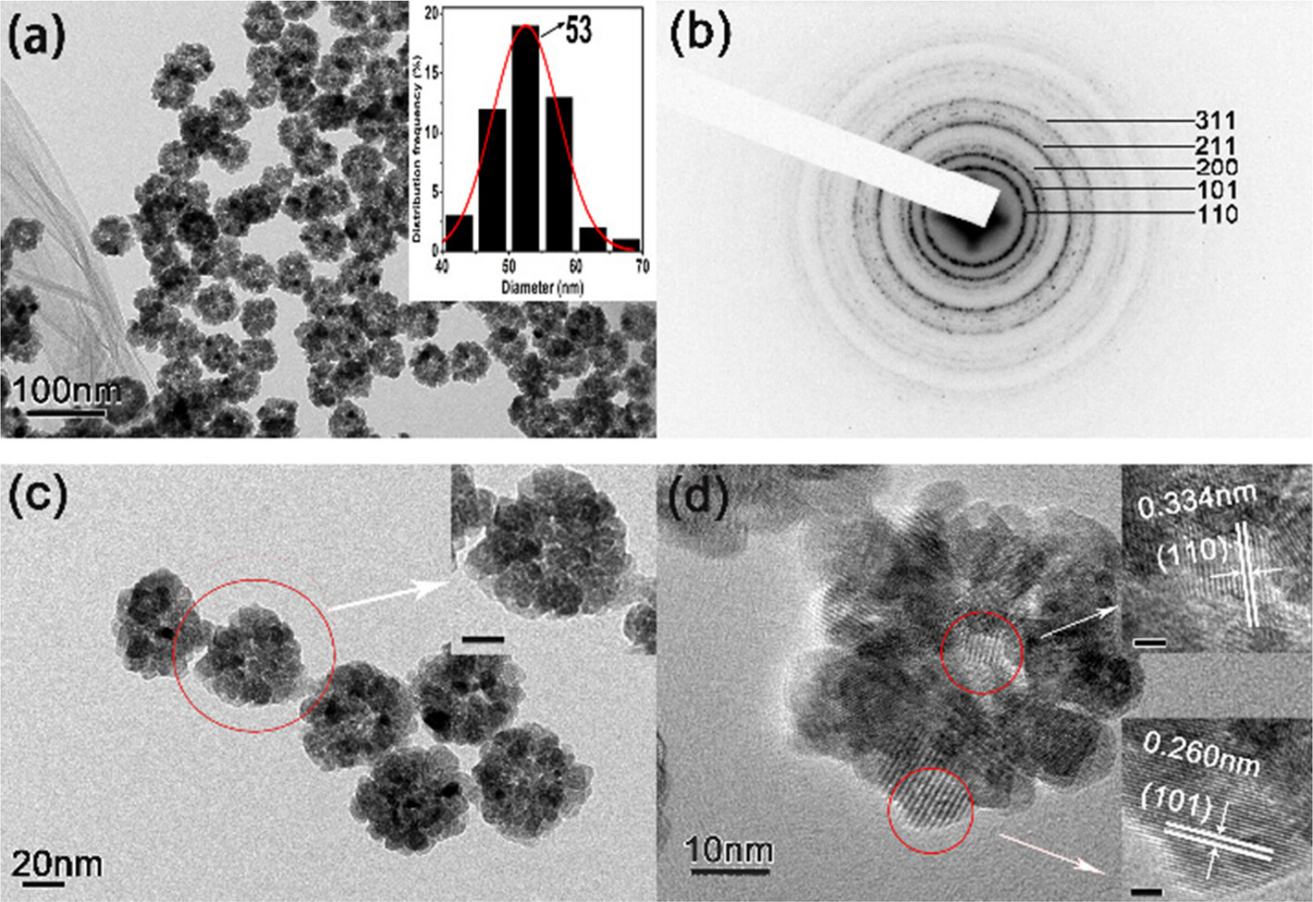
**SAED patterns and TEM images at low and high magnifications. (a)** TEM image at low magnification (the inset is the histogram of particle diameters). **(b)** SAED patterns and **(c)** TEM images at high magnification (the inset scale bar is 10 nm) of the as-prepared hollow SnO_2_ nanoparticles, and **(d)** HRTEM image of a single SnO_2_ nanoparticle (the inset scale bar is 2 nm).

The structure and morphology of the as-prepared hollow SnO_2_ nanoparticles are investigated by TEM and HRTEM. As shown in Figure 2a, the as-prepared samples mainly consist of uniform flower-like nanoparticles. The contrast (dark/bright) between the boundary and the center of the nanoparticles confirms their hollow nature. The histogram of the particle diameters (inset in Figure 2a) demonstrates that the average diameter of the as-prepared hollow SnO_2_ nanoparticles is 53 nm. The bright rings in the selected-area electron diffraction (SAED) pattern (Figure 2b) can be well indexed to the rutile-phase SnO_2_. Figure 2c shows the TEM image at high magnification of the hollow SnO_2_ nanoparticles. It can be seen that the SnO_2_ particles were consist of small SnO_2_ grains, and the surface of the SnO_2_ particles is rough, which means that the shell is incomplete and porous, not solid. This feature endows that the hollow SnO_2_ nanoparticles have high surface area. As shown in Figure 2d, the HRTEM image confirms that the SnO_2_ particles consist of small SnO_2_ grains, and their size is about 3 ~ 5 nm. From the insets of Figure 2d, there are two lattice fringes with lattice spacing of about 0.334 and 0.26 nm, which can be assigned to the (110) and (101) planes of tetragonal rutile-phase SnO_2_ nanoparticles, respectively.

**Figure 3:**
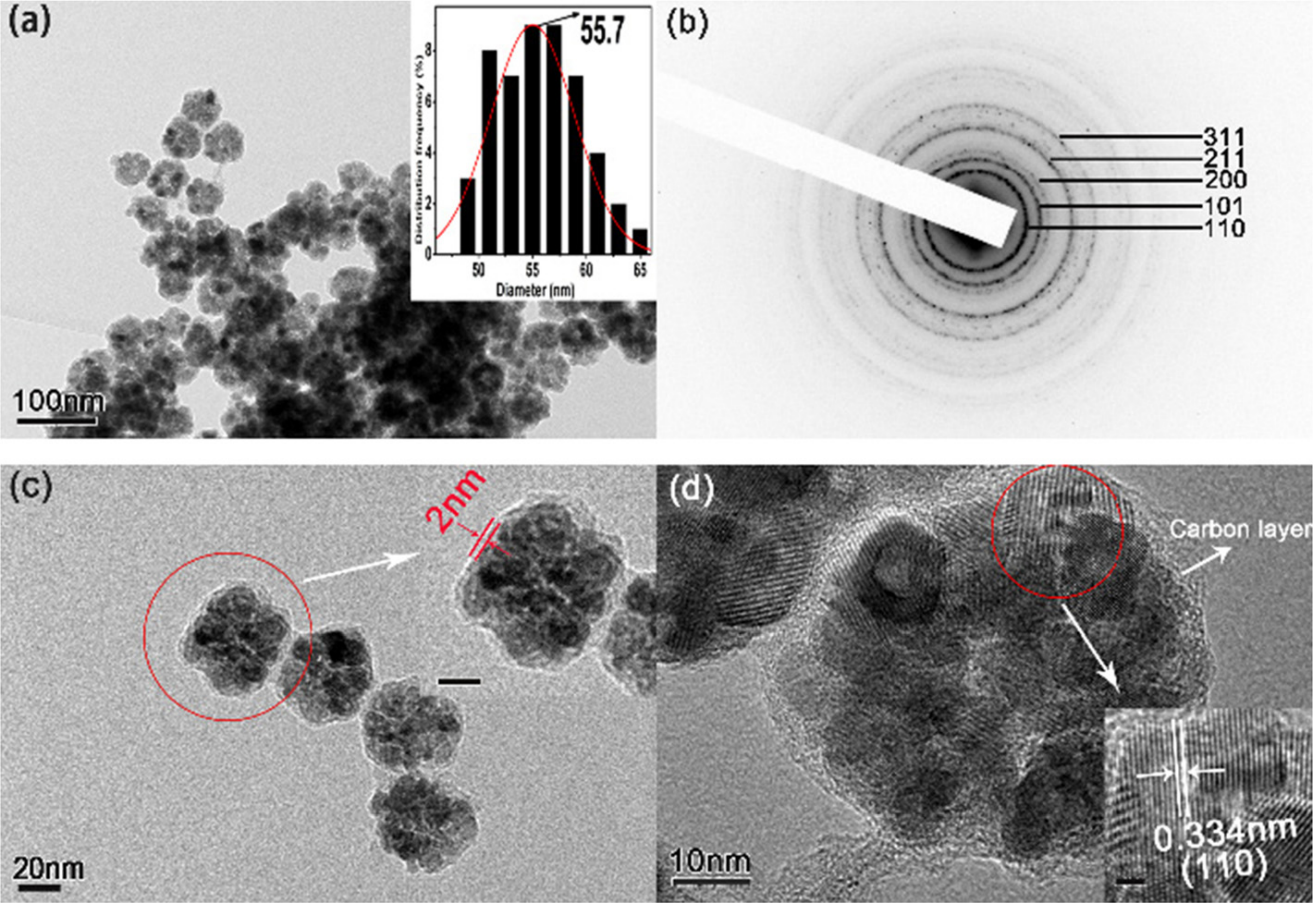
**TEM images at low and high magnifications. (a)** TEM image at low magnification (the inset is the histogram of the particle diameters). **(b)** SAED patterns and **(c)** TEM image at high magnification (the inset scale bar is 10 nm) of the as-prepared carbon-coated hollow SnO_2_ nanoparticles and **(d)** HRTEM image **(d)** of a single SnO_2_@C nanoparticle (the inset scale bar is 2 nm).

Subsequently, the morphologies of the carbon-coated hollow SnO_2_ nanoparticles (SnO_2_@C) were further studied by TEM and HRTEM. Figure 3a shows the TEM image of the SnO_2_@C nanoparticles. It can be seen that the SnO_2_@C nanoparticles still maintained a uniform morphology. The inset histogram diameters illustrate that the average diameter of SnO_2_@C nanoparticles is 55.7 nm. Compared with the naked hollow SnO_2_ nanoparticles, the thickness of the carbon coating layer is about 2 ~ 3 nm. As shown in Figure 3b, the bright rings in the SAED pattern can be well indexed to the structure of the rutile-phase SnO_2_, which demonstrate that the structure of SnO_2_ is also not change by carbon coating. From the magnified TEM images (Figure 3c), a thin carbon layer on the surface of the SnO_2_ nanoparticles can be observed clearly, and the thermal gravimetric analysis (Additional file 1: Figure S1) illustrates that about 37% of carbon has coated the SnO_2_ nanoparticles. The HRTEM image (Figure 3d) shows that the carbon layer is smooth, continuous, and has a thickness of about 2 ~ 3 nm. There are lattice fringes with lattice spacing of about 0.334 nm, which can be indexed to the (110) plane of tetragonal rutile-phase SnO_2_ nanoparticles. The above results prove that the carbon has been successfully coated on the surface of the hollow SnO_2_ nanoparticles, and the morphology is still maintained after the coating treatment.

**Figure 4:**
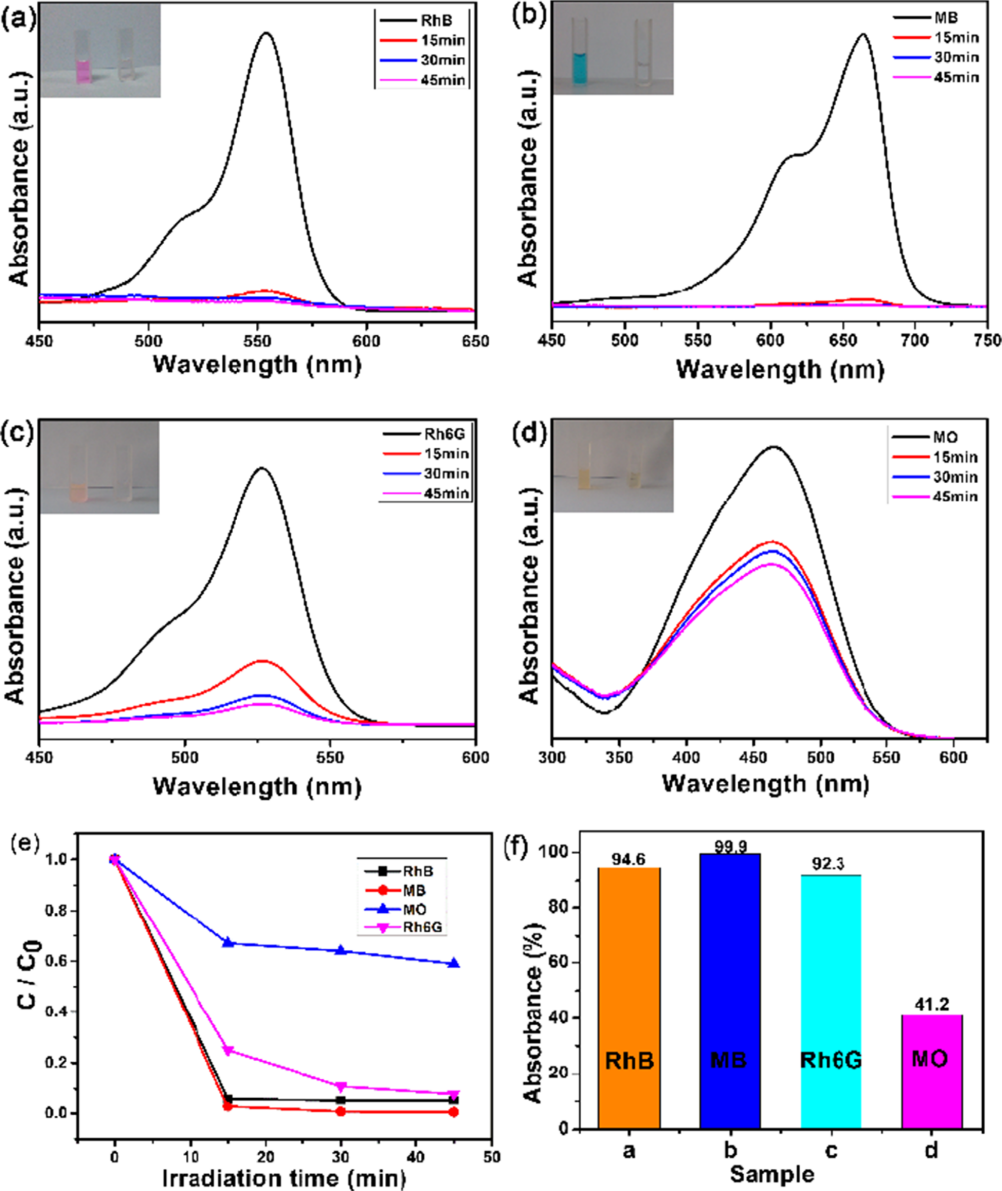
**UV-vis absorption spectra.** RhB **(a)**, MB **(b)**, Rh6G **(c)**, and MO **(d)** when the hollow SnO_2_@C nanoparticles were present at different times (the insets are the photos of their dyes before and after being treated with the as-synthesized SnO_2_@C nanoparticles). The adsorption kinetics and adsorption isotherm with the corresponding dyes **(e)** and the comparison absorbance **(f)** for the removal rate of SnO_2_@C hollow nanoparticles (the concentration of dyes is as follows: RhB 10 mg/L, MB 5 mg/L, Rh6G 5 mg/L, and MO 5 mg/L).

We also investigated the potential application of the as-synthesized carbon-coated hollow SnO_2_ nanoparticles to be used as an adsorbent in wastewater treatment. Figure 4 shows the time-dependent absorption spectra for different organic dyes (include RhB, Rh6G, MB, and MO) in 45 min, and the characteristic absorption of these dyes was used to monitor the process of adsorption. The used dyes are chemically stable and are common constituents of effluents in industries which demand an appropriate method to dispose them off. As shown in Figure 4a,b,c, we can see that the peak intensities at 554 nm for RhB, 664 nm for MB, and 525 nm for Rh6G decreased very quickly once the hollow SnO_2_@C were added. After only 45 min, these peaks became too weak to be observed, suggesting the high efficiency for removing these three dyes. Meanwhile, the insets of Figure 4a,b,c shows the change of the color of these three dyes in solution within 45 min. It can be seen that the color of the three dyes disappeared, suggesting that the chromophoric structure of RhB, MB, and Rh6G were decomposed. However, for the removal of MO, the color of the MO solutions did not disappear in 45 min (Figure 4d). This means that a part of the molecular structure of MO was not decomposed by SnO_2_@C and remained in the solution.

Figure 4e,f further confirms that the removal rate of RhB (10 mg/L) can reach to 94.6%. The results reveal that the as-prepared hollow SnO_2_@C nanoparticles exhibit excellent removal performance for RhB dyes. Meanwhile, the hollow SnO_2_@C nanoparticles also showed a good removal performance for MB and Rh6G (5 mg/L); the removal rate can reach to 99.9% and 92.3%, respectively. However, for the MO dyes (5 mg/L), the removal rate can only reach to 41.2%, because the chromophoric structure of MO dye is different from those of RhB and MB, and this will cause a different electrostatic interaction capacity between functional groups of carbon and dye molecules [18-20]. The above results illustrate that the as-obtained hollow SnO_2_@C nanoparticles exhibit a good dye removal performance.

**Figure 5:**
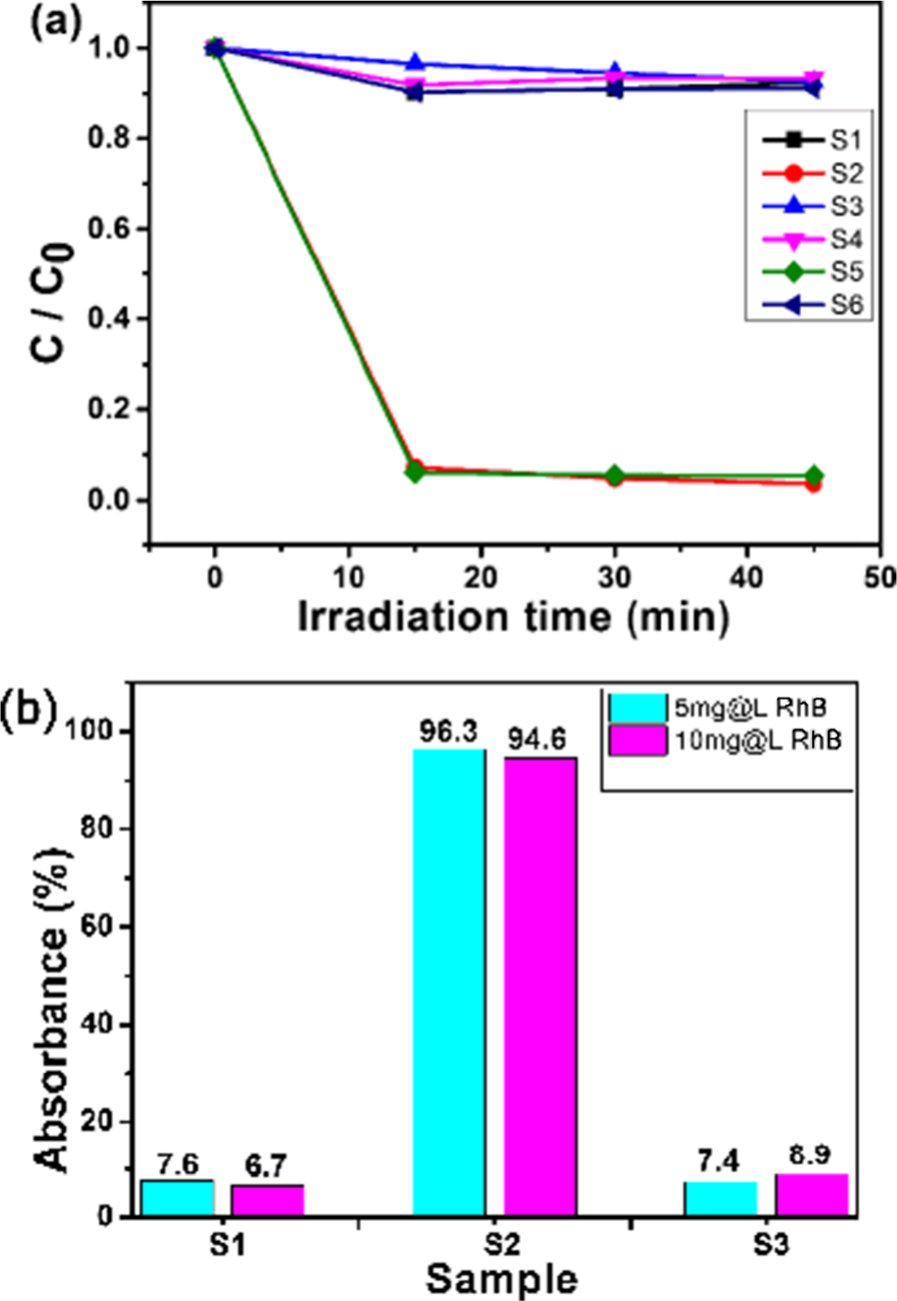
**Adsorption kinetics and removal rate. (a)** Adsorption kinetics and adsorption isotherm with the corresponding percentage removal of RhB at two different initial concentrations (*C*) with a contact time of 45 min (S1 and S4 are naked hollow SnO_2_ nanoparticles, S2 and S5 are hollow SnO_2_@C nanoparticles, and S3 and S6 are commercial SnO_2_ nanoparticles; the *C*_RhB_ for S1 to S3 is 5 mg/L, and the *C*_RhB_ for S4 to S6 is 10 mg/L). **(b)** The comparison of the removal rate of the different samples (S1: hollow SnO_2_, S2: hollow SnO_2_@C nanoparticles, S3: commercial SnO_2_).

To further study the dye removal abilities of the as-prepared hollow SnO_2_@C nanoparticles, the dye removal performance of naked hollow SnO_2_ nanoparticles and commercial SnO_2_ nanoparticles (average size is 70 nm) was measured for comparison. Figure 5a shows the time-dependent adsorption kinetics of the samples at different initial RhB dye concentrations. Obviously, among all the samples, the hollow SnO_2_@C nanoparticles (samples S2 and S5) exhibit the fastest absorption abilities. As shown in Figure 5b, the removal rate of the hollow SnO_2_@C nanoparticles (S2) is highest among the three samples and can reach to 96.3% and 94.6% for the RhB dye with different concentration of 5 and 10 mg/L, respectively, which is much higher than that of S1 (naked hollow SnO_2_, 7.6% and 6.7%) and S3 (commercial SnO_2_, 7.4% and 8.9%). The above results demonstrate that carbon coating can significantly enhance the dye removal abilities. As a comparison, the measured results of the removal performance experiment of carbon sphere and hydrochloric acid-treated SnO_2_@C nanoparticles (SnO_2_ has been removed) are shown in Additional file 1: Figures S2 and S3. The results show that the as-prepared hollow SnO_2_@C nanoparticles' removal dye performance is better than those of pure carbon materials.

**Figure 6:**
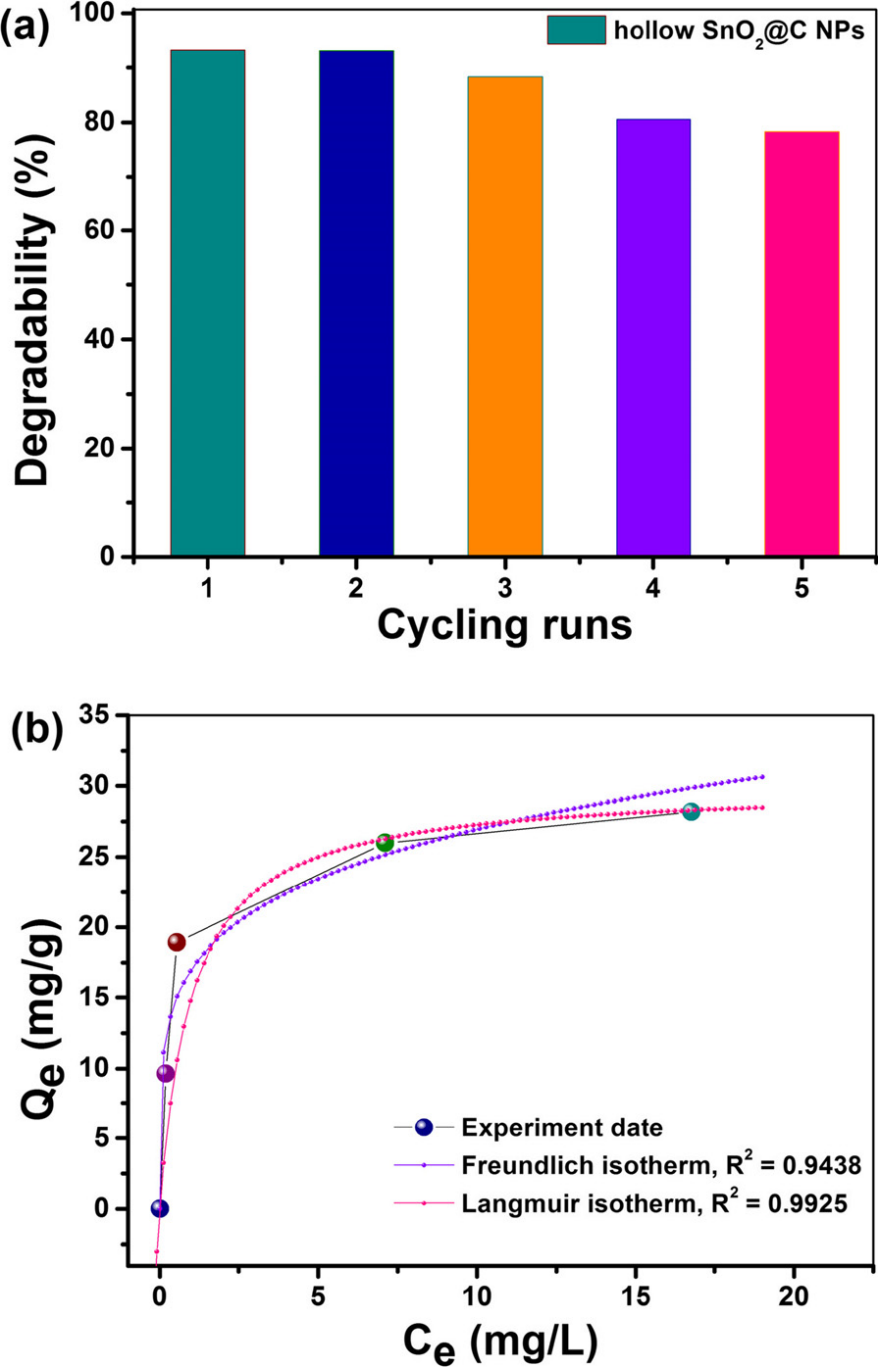
**Reutilization properties.** Removal performance under five cycles **(a)** and isotherms **(b)** for RhB adsorption on the as-obtained hollow SnO_2_@C nanoparticles.

Subsequently, the stability of the as-prepared hollow SnO_2_@C nanoparticles has been further investigated by recycling the removal for RhB, and the results are shown in Figure 6a. The hollow SnO_2_@C nanoparticles exhibited a good removal dye activity and stability; the degradation rate of RhB solution was found to be more than 78% after 5 cycles. As shown in Figure 6b and Additional file 1: Figure S4, the adsorption capacity for RhB increased with the different RhB concentrations. The maximum adsorption capacity in the concentration range studied is 28.2 mg/g for RhB. The amount of the dye adsorbed was calculated using the equation: *Q*_e_ = (*C*_0_ − *C*_e_) *V*/*m*, where *Q*_e_ (mg/g) is the amount of RhB adsorbed onto the adsorbent at equilibrium, *C*_0_ (mg/L) and *C*_e_ (mg/L) are the initial and equilibrated RhB concentrations, respectively, *V* (*L*) is the volume of solution added, and *m* (g) is the mass of the adsorbent. Figure 6b shows the isotherms for RhB adsorption on the as-obtained SnO_2_@C nanoparticles. It can be found that the regression coefficient *R*^2^ obtained from the Langmuir model is much higher than that of from the Freundlich model (0.9925 > 0.9438), suggesting the Langmuir model fits better with the experimental data [21].

**Figure 7:**
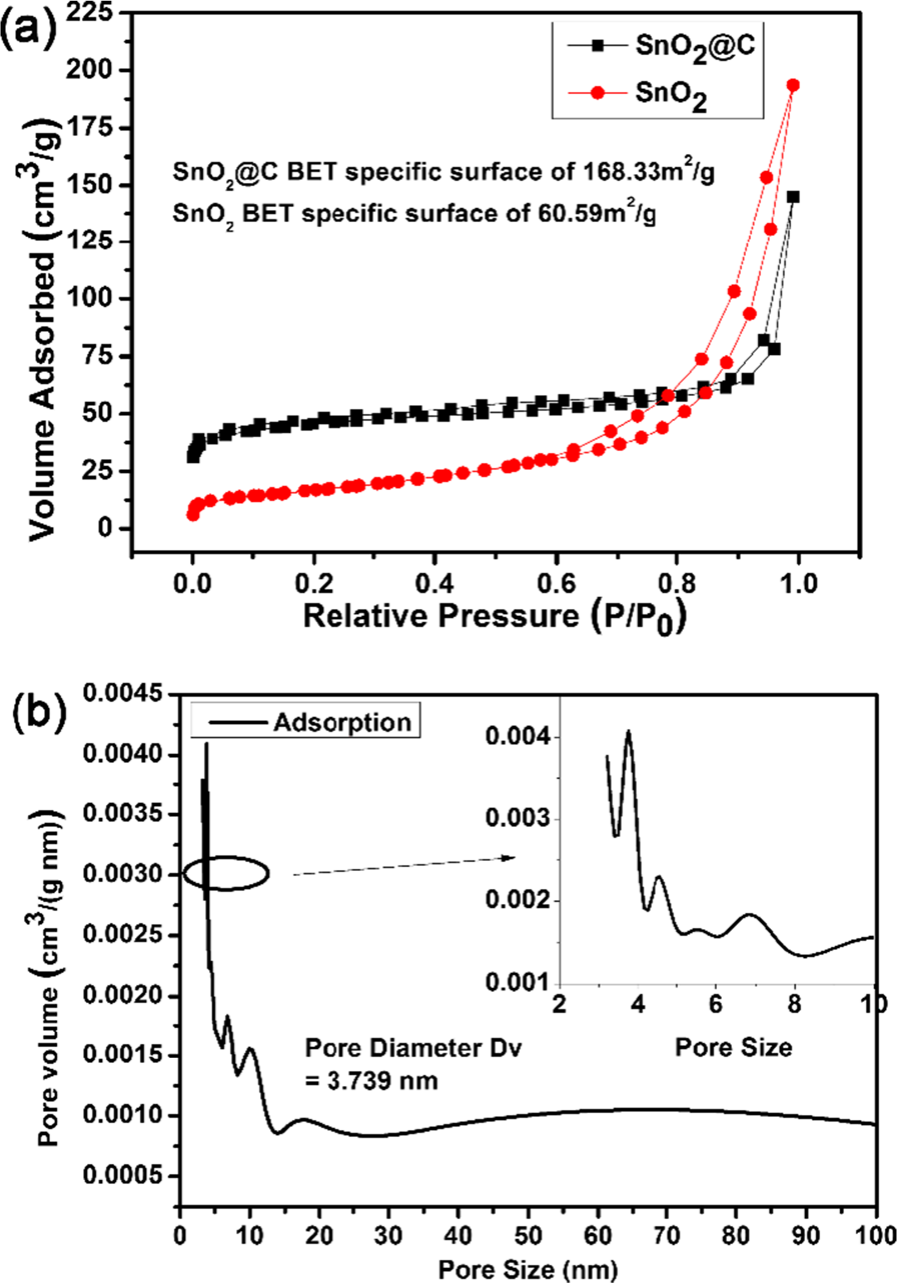
**Nitrogen adsorption-desorption isotherms and pore size distribution. (a)** Nitrogen adsorption-desorption isotherms of the as-synthesized SnO_2_ and hollow SnO_2_@C nanoparticles. **(b)** The pore size distribution of the hollow SnO_2_@C nanoparticles.

**Figure 8:**
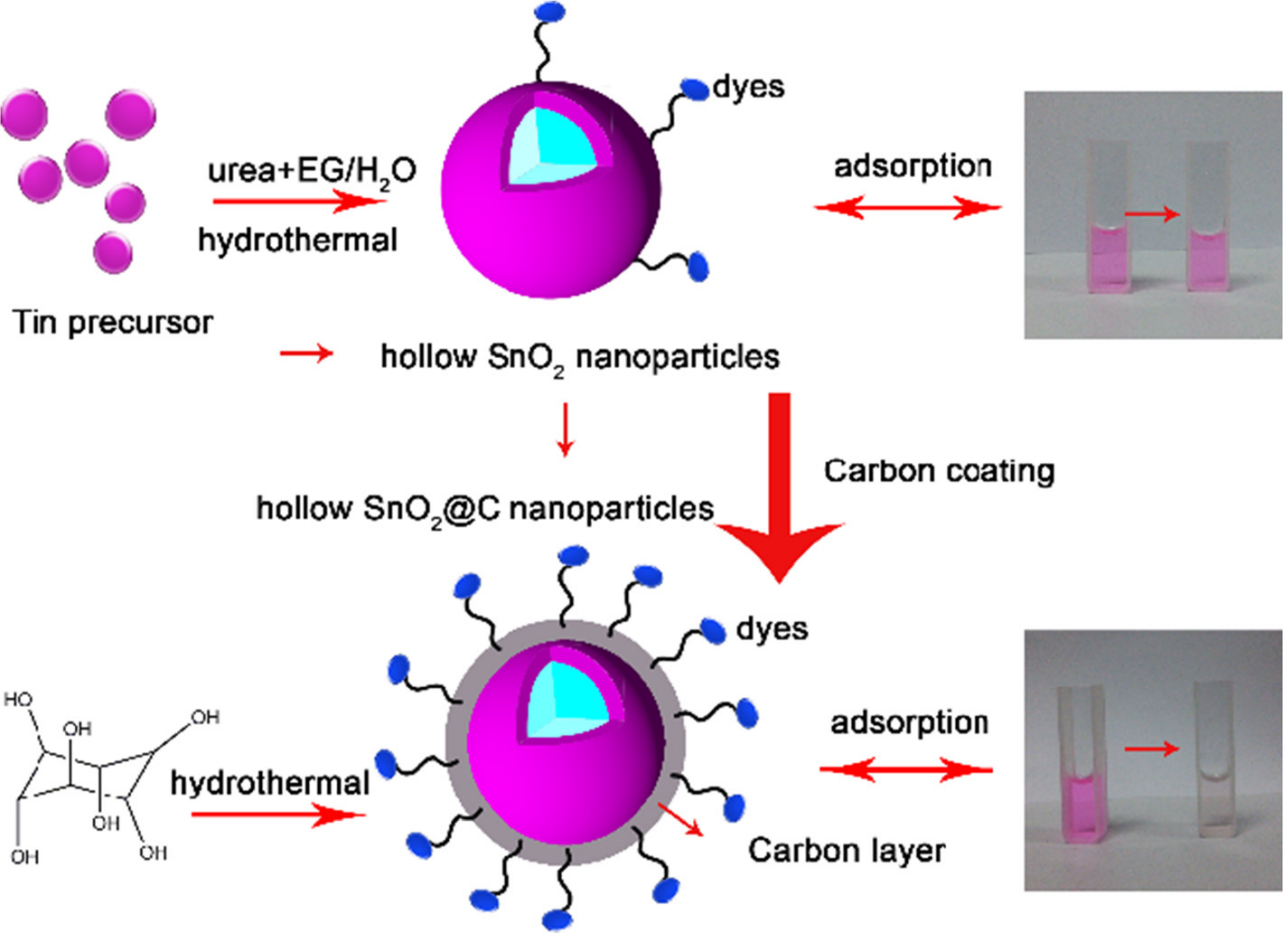
Schematic illustration of synthesis and dye removal processes.

To avoid the photocatalytic effect of SnO_2_ and SnO_2_@C nanoparticles, the dye removal tests are carried out in a dark environment. And the results reveal that the carbon coating can enhance the absorption abilities. To illustrate the reason, the nitrogen adsorption isotherms of the hollow SnO_2_ and SnO_2_@C nanoparticles have been measured and shown in Figure 7. The BET surface areas of the hollow SnO_2_ and SnO_2_@C nanoparticles are 60.59 and 168.33 m^2^/g, respectively. Both samples exhibit the type IV isotherms with a distinct hysteresis loop at the relative pressure *P*/*P*_*0*_ ranging from 0.5 to 0.8. Clearly, the carbon coating will greatly enhance the surface area, which can be the main reason of significant enhanced dye removal performance of hollow SnO_2_@C nanoparticles. The large number and array of different functional groups on the carbon layers (e.g., carboxylic, hydroxyl, carbonyl) implied the existence of many types of adsorbent-solute interaction [22]. Additionally, carbon coating has made the covalent bond interaction with hexagonal structure, which has a -π structure properties of aromatic ring, easy to interact with conjugated double bonds. And some of the dye structure have conjugated double bonds and easy to be adsorbed by the coating carbon [23]. As shown in Figure 8, the hollow SnO_2_@C nanoparticles can capture more dye molecules due to the introduced carbon layer. Indeed, relatively larger amount of water and hydroxyl groups can be adsorbed on the surface by hydrothermal process [24]. The surface chemistry of the adsorbents plays a major role in the adsorption. The adsorption of the reactive dye on carbon is favored, mainly due to the dispersive interactions between the delocalized π electrons of the carbon materials and the free electrons of the dye molecules [20]. The functional groups on the hollow SnO_2_@C nanoparticles' surface acted as a negative potential that provides a weak electrostatic interaction between the organic dyes and the hollow SnO_2_@C nanoparticles.

## Conclusions

In summary, hollow SnO_2_@C nanoparticles have been synthesized on a large scale through a facile hydrothermal method. The as-prepared hollow SnO_2_@C nanoparticles show excellent adsorption capacity toward RhB, MB, and Rh6G dyes in aqueous solutions. Compared with the naked hollow SnO_2_ and commercial SnO_2_ nanoparticles, the adsorption capacity showed about an 89% improvement for RhB organic dye. The porous carbonaceous shells coated on the surface of hollow SnO_2_ nanoparticles greatly enhanced the specific area, which provides more active sites for dye adsorption. Owing to their unique hollow structures, high surface areas and low cost, the as-obtained hollow SnO_2_@C nanoparticles are potentially applicable in wastewater treatment. Accordingly, it may be concluded that the developed SnO_2_@C is an efficient method for the decolorization of RhB, MB, and Rh6G dyes.

## Competing interests

The authors declare that they have no competing interests.

## Authors’ contributions

SY carried out the absorbance studies and drafted the manuscript. ZW, BZ, and JP participated in the dye removal analysis. LPH, ML, LS, and QT did the fabrication and characterization experiments. WW and HZ analyzed the results and participated in its design and coordination. All authors read and approved the final manuscript.

## Supplementary Material

Additional file 1 **Supporting information.** Thermal gravimetric analysis, UV-vis absorption spectra of dyes, adsorption kinetics, and the effect of RhB dye equilibrium concentrations of SnO2@C nanoparticles. Click here for file

## References

[B1] Lei W Portehault D Liu D Qin S Chen Y Porous boron nitride nanosheets for effective water cleaning Nat Commun 2013 9 1777 23653189 10.1038/ncomms2818

[B2] Rafatullah M Sulaiman O Hashim R Ahmad A Adsorption of methylene blue on low-cost adsorbents: a review J Hazard Mater 2010 9 70 80 10.1016/j.jhazmat.2009.12.047 20044207

[B3] Hsiu-Mei C Ting-Chien C San-De P Chiang HL Adsorption characteristics of Orange II and Chrysophenine on sludge adsorbent and activated carbon fibers J Hazard Mater 2009 9 1384 1390 10.1016/j.jhazmat.2008.04.102 18539385

[B4] Tseng RL Wu FC Juang RS Liquid-phase adsorption of dyes and phenols using pinewood-based activated carbons Carbon 2003 9 487 495 10.1016/S0008-6223(02)00367-6

[B5] Namasivayam C Kavitha D Removal of Congo red from water by adsorption onto activated carbon prepared from coir pith, an agricultural solid waste Dyes Pigments 2002 9 47 58 10.1016/S0143-7208(02)00025-6

[B6] Guo Y Yang S Fu W Qi J Li R Wang Z Xu H Adsorption of malachite green on micro-and mesoporous rice husk-based active carbon Dyes Pigments 2003 9 219 229 10.1016/S0143-7208(02)00160-2

[B7] Hameed B Din AM Ahmad A Adsorption of methylene blue onto bamboo-based activated carbon: kinetics and equilibrium studies J Hazard Mater 2007 9 819 825 10.1016/j.jhazmat.2006.07.049 16956720

[B8] Zhu T Chen JS Lou XW Highly efficient removal of organic dyes from waste water using hierarchical NiO spheres with high surface area J Phys Chem C 2012 9 6873 6878 10.1021/jp300224s

[B9] Mou F Guan J Ma H Xu L Shi W Magnetic iron oxide chestnutlike hierarchical nanostructures: preparation and their excellent arsenic removal capabilities ACS Appl Mater Interfaces 2012 9 3987 3993 10.1021/am300814q 22796758

[B10] Mou F Guan J Xiao Z Sun Z Shi W Fan X Solvent-mediated synthesis of magnetic Fe_2_O_3_ chestnut-like amorphous-core/γ-phase-shell hierarchical nanostructures with strong As(v) removal capability J Mater Chem 2011 9 5414 5421 10.1039/c0jm03726e

[B11] Liu QC Ma DK Hu YY Zeng YW Huang SM Various bismuth oxyiodide hierarchical architectures: alcohothermal-controlled synthesis, photocatalytic activities, and adsorption capabilities for phosphate in water ACS Appl Mater Interfaces 2013 9 11927 11934 10.1021/am4036702 24138056

[B12] Fan W Gao W Zhang C Tjiu WW Pan J Liu T Hybridization of graphene sheets and carbon-coated Fe_3_O_4_ nanoparticles as a synergistic adsorbent of organic dyes J Mater Chem 2012 9 25108 25115 10.1039/c2jm35609k

[B13] Li B Cao H Yin G Mg(OH)_2_@ reduced graphene oxide composite for removal of dyes from water J Mater Chem 2011 9 13765 13768 10.1039/c1jm13368c

[B14] Duan F Dong W Shi D Chen M Template-free synthesis of ZnV_2_O_4_ hollow spheres and their application for organic dye removal Appl Surf Sci 2011 9 189 195 10.1016/j.apsusc.2011.08.029

[B15] Wu W Xiao X Zhang S Li H Zhou X Jiang C One-pot reaction and subsequent annealing to synthesis hollow spherical magnetite and maghemite nanocages Nanoscale Res Lett 2009 9 926 931 10.1007/s11671-009-9342-6 20596278 PMC2894336

[B16] Lou XWD Archer LA Yang Z Hollow micro-/nanostructures: synthesis and applications Adv Mater 2008 9 3987 4019 10.1002/adma.200800854

[B17] Wu W Zhang S Zhou J Xiao X Ren F Jiang C Controlled synthesis of monodisperse sub-100 nm hollow SnO_2_ nanospheres: a template- and surfactant-free solution-phase route, the growth mechanism, optical properties, and application as a photocatalyst Chem Eur J 2011 9 9708 9719 10.1002/chem.201100694 21735499

[B18] Vinu R Madras G Environmental remediation by photocatalysis J Indian Inst Sci 2010 9 189 230

[B19] Dutta S Sarkar S Ray C Pal T Benzoin derived reduced graphene oxide (rGO) and its nanocomposite: application in dye removal and peroxidase-like activity RSC Advances 2013 9 21475 21483 10.1039/c3ra44069a

[B20] Figueiredo J Sousa J Orge C Pereira M Orfao J Adsorption of dyes on carbon xerogels and templated carbons: influence of surface chemistry Adsorption 2011 9 431 441 10.1007/s10450-010-9272-8

[B21] Kyzas GZ Kostoglou M Lazaridis NK Relating interactions of dye molecules with chitosan to adsorption kinetic data Langmuir 2010 9 9617 9626 10.1021/la100206y 20302276

[B22] Al-Ghouti MA Li J Salamh Y Al-Laqtah N Walker G Ahmad MNM Adsorption mechanisms of removing heavy metals and dyes from aqueous solution using date pits solid adsorbent J Hazard Mater 2010 9 510 520 10.1016/j.jhazmat.2009.11.059 19959281

[B23] Sun H Cao L Lu L Magnetite/reduced graphene oxide nanocomposites: one step solvothermal synthesis and use as a novel platform for removal of dye pollutants Nano Res 2011 9 550 562 10.1007/s12274-011-0111-3

[B24] Baiju KV Shukla S Biju S Reddy MLP Warrier KGK Morphology-dependent dye-removal mechanism as observed for anatase-titania photocatalyst Catal Lett 2009 9 663 671 10.1007/s10562-009-0010-3

