# Supplementary material for: Significantly enhanced dye removal performance of hollow tin oxide nanoparticles via carbon coating in dark environment and study of its mechanism
Source: Nanoscale Res Lett. 2014 Aug 28;9(1):442. doi: 10.1186/1556-276X-9-442 (PMC4150862; doi:10.1186/1556-276X-9-442)
Supplement: Additional file 1 — Supporting information. Thermal gravimetric analysis, UV-vis absorption spectra of dyes, adsorption kinetics, and the effect of RhB dye equilibrium concentrations of SnO2@C nanoparticles. [file 1556-276X-9-442-S1.docx]

Supporting Information

Significantly Enhanced Dyes Removal Performance of Hollow Tin Oxide Nanoparticles via Carbon Coating in Dark Environment and the Mechanism Study

Shuanglei Yang^1, 2^, Zhaohui Wu^4^, LanPing Huang^1^, Banghong Zhou^1^, Mei Lei^3^, Lingling Sun^3^, Qingyong Tian^3^, Jun Pan^1^, Wei Wu^2^* and Hongbo Zhang^1^^[[1]](#footnote-1)^

^1^State Key Laboratory for Powder Metallurgy, Central South University, Changsha 410083, P.R. China.

^3^Laboratory of Printable Functional Nanomaterials and Printed Electronics, School of Printing and Packaging, Wuhan University, Wuhan 430072, P.R. China.

^3^Key Laboratory of Artificial Micro and Nano-structures of Ministry of Education, School of Physics and Technology, Wuhan University, Wuhan 430072,P.R. China.

^4^Department of Chemical Engineering, Kyung Hee University, Seocheon-Dong, Giheung-Gu, 446-701 Yongin, Korea





**Figure S1** Thermal gravimetric analysis of as-obtained hollow SnO_2_@C nanoparticles.


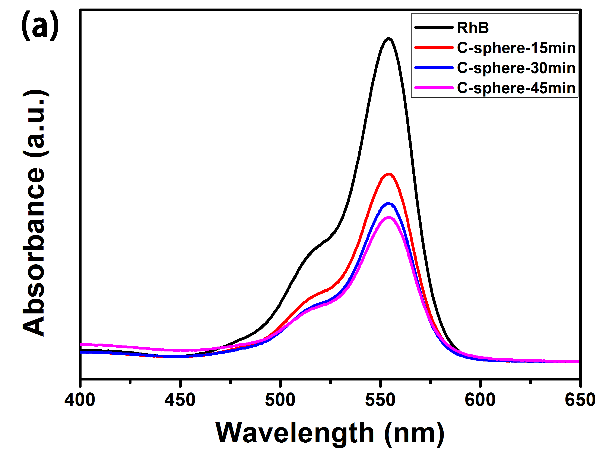

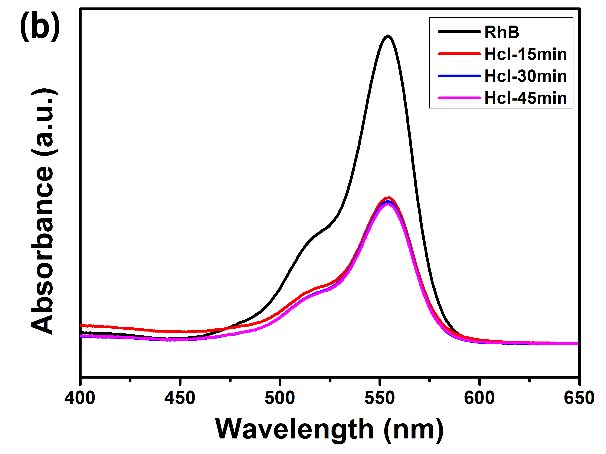


**Figure S2** UV-vis absorption spectra of dyes in the presence of as-prepared carbon sphere (a), SnO_2_@C treatment by hydrochloric acid (b) at different time intervals.





**Figure S3** Adsorption kinetics and adsorption isotherm with the corresponding percentage removal of RhB





**Figure S4** The effect of RhB dye equilibrium concentrations of as-obtained hollow SnO_2_@C nanoparticles.

1. To whom correspondence should be addressed. Tel: +86-27-68778529. Fax: +86-27-68778433. E-mail: [weiwu@whu.edu.cn](mailto:weiwu@whu.edu.cn) (W. Wu), [13973183375@139.com](mailto:13973183375@139.com) (H. B. Zhang). [↑](#footnote-ref-1)
